# Ventilator-associated Pneumonia and MRSA ST398, Italy

**DOI:** 10.3201/eid1604.091584

**Published:** 2010-04

**Authors:** Caterina Mammina, Cinzia Calà, Maria R.A. Plano, Celestino Bonura, Antonietta Vella, Rachele Monastero, Daniela M. Palma

**Affiliations:** University of Palermo, Palermo, Italy (C. Mammina, C. Calà, M.R.A. Plano, C. Bonura, A. Vella); ARNAS Ospedale Civico Di Cristina e Benfratelli, Palermo (R. Monastero, D.<. Palma)

**Keywords:** Methicillin-resistant Staphylococcus aureus, MRSA, ST398, ventilator-associated pneumonia, expedited, animals, Italy, letter

**To the Editor:** Methicillin-resistant *Staphylococcus aureus* (MRSA) sequence type (ST)398 has become increasingly common in livestock, particularly pigs, in some countries in Europe, such as Spain and Germany (*1*). In Italy, prevalences as high as 14% and 21.6% in pig-breeding facilities and meat-processing sites, respectively, have been recently reported (*1*).

Possible association of MRSA in animals with infection in humans has been investigated. One study showed a strong relationship between contact with pigs or calves and carriage by persons having direct contact with animals and families of persons who handle animals (*2*). Moreover, an MRSA prevalence >11.9% has been described by de Boer et al. (*3*) in meat, with 85% of isolates belonging to the ST398 lineage.

MRSA ST398 has been described as a lineage with limited virulence and ability to spread between humans, but severe clinical manifestations, such as wound infections and endocarditis, have been recently attributed to this clone (*1*,*4*). Cases of nosocomial ventilator-associated pneumonia have also been reported in Germany (*1*). Moreover, an outbreak of infection with MRSA ST398 occurred in a surgical ward of a hospital in the Netherlands in 2007 (*5*).

MRSA ST398 is an infrequent cause of human infections in Italy. No isolates belonged to this lineage in 2 studies of MRSA in Italy during 2006–2007 (*6*) or in hospitals during 1990–2007 (*7*). Only 1 invasive infection has been recently reported in a pig farm worker (*8*). We report a case of ventilator-associated pneumonia caused by MRSA ST398 in a patient in Palermo, Italy. The patient and his household members did not report any exposure to companion or livestock animals.

The case-patient was a 78-year-old man admitted to a cardiac intensive care unit (ICU) of ARNAS Ospedale Civico Di Cristina e Benfratelli in Palermo on January 31, 2009, because of a recent history of unstable angina pectoris and acute anemia caused by duodenal ulcers. After cardiocirculatory arrest, he was transferred to a general ICU on February 3. The patient had type 2 diabetes and ischemic-hypertensive cardiomyopathy. MRSA nasal colonization at admission was not investigated because the patient lacked risk factors for screening at admission, e.g., antimicrobial drug therapy, hospitalization for >48 hours or time in a long-term care facility within the past 6 months, need for long-term nursing care, presence of indwelling devices, or chronic skin lesions.

The clinical course of the patient’s illness was characterized by serious hemodynamic instability and difficulty in weaning from mechanical ventilation. Two bronchial aspirate specimens were cultured on February 4 and 9, when he was being treated with a third-generation cephalosporin (ceftriaxone). These cultures showed *Staphylococcus epidermidis* and *S*. *saprophyticus*. On the 14th day in the ICU, clinical signs of ventilator-associated pneumonia developed in the patient. He had increased sputum production, fever (38.8°C), leukocytosis, and infiltrates were seen on a chest radiograph.

Empiric antimicrobial drug therapy with glycopeptides and a β-lactam/β-lactamase inhibitor combination was started. Culture of bronchial secretions yielded MRSA that was susceptible to glycopeptides, rifampin, linezolid, macrolides, and sulfamethoxazole and resistant to fluoroquinolones and tetracyclines. Three days later, linezolid was given, but the patient died after an acute myocardial infarction.

The isolate was identified genetically by *mec*A PCR. It was not typeable by pulsed-field gel electrophoresis after digestion with *Sma*I, negative for Panton-Valentine leukocidin, and carried staphylococcal cassette chromosome *mec* (SCC*mec*) type IVa (*9*). Multilocus sequence typing, performed according to a recommended procedure (http://saureus.mlst.net/misc/info.asp), identified the isolate as ST398.

A 1-year epidemiologic survey on MRSA isolates from 4 general hospitals in Palermo, which had begun on February 2009, did not identify any MRSA isolate carrying SCC*mec* type IV or V in patients admitted to the ICU until September 2009. However, colonization or infection by MRSA ST398 in the ICU patients before the study period could not be ruled out. Although an MRSA screening policy for the ICU staff members was not being carried out, a nosocomial chain of transmission appeared to be unlikely.

Our results indicate that a new zoonotic clone of MRSA is emerging as a potential cause of serious human infections. Screening at hospital admission would likely help efforts to determine whether exposure to pet animals and livestock had occurred. However, the absence of specific exposure to zoonotic clonal lineages, as in our case-patient, is a matter of concern in terms of screening and contact tracing policy for MRSA infections. Prevalence of MRSA and distribution of MRSA sequence types in livestock in Italy are not known. However, surveys of foods of animal (pig) origin have showed an MRSA prevalence of 3.7% (*1*,*10*). In view of the low prevalence of MRSA ST398 in patients with no exposure to animals, food products currently seem to play a negligible role. However, this clone is likely spreading because of the large animal reservoir of ST398 and the global market for meat and livestock. The changing epidemiology of MRSA indicates that collaborative surveillance plans integrating human and animal information should be increased.

## References

[1] European Food Safety Authority. Analysis of the baseline survey on the prevalence of methicillin-resistant *Staphylococcus aureus* (MRSA) in holdings with breeding pigs, in the EU, 2008. European Food Safety Authority Journal. 2009;7:1376. 10.2903/j.efsa.2022.7620 PMC9579990 36267542

[2] van Loo I, Huijsdens X, Tiemersma E, de Neeling A, van de Sande-Bruinsma N, Beaujean D, Emergence of methicillin-resistant *Staphylococcus aureus* of animal origin in humans. Emerg Infect Dis. 2007;13:1834–9. 18258032 10.3201/eid1312.070384 PMC2876750

[3] de Boer E, Zwartkruis-Nahuis JT, Wit B, Huijsdens XW, de Neeling AJ, Bosch T, Prevalence of methicillin-resistant *Staphylococcus aureus* in meat. Int J Food Microbiol. 2009;134:52–6. 10.1016/j.ijfoodmicro.2008.12.007 19144432

[4] Ekkelenkamp MB, Sekkat M, Carpaij N, Troelstra A, Bonten MJ. Endocarditis due to methicillin-resistant *Staphylococcus aureus* originating from pigs [in Dutch]. Ned Tijdschr Geneeskd. 2006;150:2442–7. 17131705

[5] Wulf MW, Markestein A, van der Linden FT, Voss A, Klaassen C, Verduin CM. First outbreak of methicillin-resistant *Staphylococcus aureus* ST398 in a Dutch hospital, June 2007. Euro Surveill. 2008;13:8051. 18445406

[6] Campanile F, Bongiorno D, Borbone S, Stefani S. Hospital-associated methicillin-resistant *Staphylococcus aureus* (HA-MRSA) in Italy. Ann Clin Microbiol Antimicrob. 2009;8:22. 10.1186/1476-0711-8-22 19552801 PMC2708121

[7] Marchese A, Gualco L, Maioli E, Debbia E. Molecular analysis and susceptibility patterns of methicillin-resistant *Staphylococcus aureus* (MRSA) strains circulating in the community in the Ligurian area, a northern region of Italy: emergence of USA300 and EMRSA-15 clones. Int J Antimicrob Agents. 2009;34:424–8. 10.1016/j.ijantimicag.2009.06.016 19651493

[8] Pan A, Battisti A, Zoncada A, Bernieri F, Boldini M, Franco A, Community-acquired methicillin-resistant *Staphylococcus aureus* ST398 infection, Italy. Emerg Infect Dis. 2009;15:845–7. 10.3201/eid1505.081417 19402995 PMC2687035

[9] Zhang K, McClure JA, Elsayed S, Louie T, Conly JM. Novel multiplex PCR assay for characterization and concomitant subtyping of staphylococcal cassette chromosome *mec* types I to V in methicillin-resistant *Staphylococcus aureus.* J Clin Microbiol. 2005;43:5026–33. 10.1128/JCM.43.10.5026-5033.2005 16207957 PMC1248471

[10] Normanno G, Corrente M, La Salandra G, Dambrosio A, Quaglia NC, Parisi A, Methicillin-resistant *Staphylococcus aureus* (MRSA) in foods of animal origin product in Italy. Int J Food Microbiol. 2007;117:219–22. 10.1016/j.ijfoodmicro.2007.04.006 17533002

